# Examining the Role of Item Sequencing of the Interpersonal Needs Questionnaire‐15

**DOI:** 10.1111/sltb.70133

**Published:** 2026-08-16

**Authors:** Sarah Sparks, Julianne E. Cary, Kirsten Christensen, Sean M. Mitchell, Sarah L. Brown, Sarah E. Victor

**Affiliations:** ^1^ Department of Psychological Sciences Texas Tech University Lubbock Texas USA; ^2^ Department of Psychology Florida State University Tallahassee Florida USA

**Keywords:** interpersonal theory of suicide, item sequence, perceived burdensomeness, psychometrics, suicide ideation, thwarted belongingness, thwarted interpersonal needs

## Abstract

**Background:**

The Interpersonal Needs Questionnaire (INQ) is a 15‐item self‐report assessment of perceived burdensomeness (PB) and thwarted belongingness (TB), conceptualized as proximal risk factors for suicide ideation (SI). The INQ‐15 has strong psychometric properties; however, PB has demonstrated more robust associations with SI than TB.

**Method:**

To assess the possible influence of INQ‐15 item sequencing, confirmatory factor analyses (CFAs) were conducted to examine the two‐factor structure of the INQ‐15 (TB and PB) among participants (*N* = 600, 50.5% women, 66.8% White, M_age_ = 38.8 years) recruited via Amazon Mechanical Turk who were randomized into three item sequence conditions (standard‐order, *n* = 192; reversed‐order, *n* = 210; random‐order, *n* = 198).

**Results:**

Across all conditions, TB items 9, 11, and 12 raised model‐fit concerns. Model fit improved after correlating residuals and removing those items across conditions. The TB‐PB correlations were weaker than those reported in prior literature, even after removing those items. The conditions and models did not differ in their associations with TB and PB, or with SI‐related variables.

**Conclusions:**

Removing TB items 9, 11, and 12 could optimize the INQ‐15 factor structure and improve its concurrent validity; however, replication in additional populations and with other sampling methods is necessary.

## 1 Introduction

The interpersonal theory of suicide (ITS; Joiner 2005; Van Orden et al. 2010), a prominent theory of suicidal behavior that has been supported by meta‐analytic findings (Chu et al. 2017), posits that thwarted interpersonal needs are key proximal risk factors for passive and active suicide ideation (SI). Thwarted interpersonal needs are specified as the constructs thwarted belongingness (TB; indicated by loneliness and the lack of reciprocal caring relationships) and perceived burdensomeness (PB; indicated by self‐hate and feelings of liability to the extent that one's death is worth more than one's life to others; Van Orden et al. 2010). When either TB or PB is present, passive SI (e.g., “I wish I were dead”) may develop. When an individual simultaneously experiences both TB and PB, and hopelessness about these two states changing, active SI may emerge (e.g., “I want to kill myself”; Van Orden et al. 2010).

The Interpersonal Needs Questionnaire (INQ; Van Orden et al. 2012) is a 15‐item self‐report measure designed to assess TB and PB, and it is the most widely used assessment of these constructs in tests of the ITS (Chu et al. 2017). Although there are other versions of the INQ, the INQ‐15 has demonstrated the most consistently strong psychometric properties (Hill et al. 2015; Van Orden et al. 2012). The INQ‐15 has two subscales (i.e., TB [9‐item scale] and PB [6‐item scale]), which are supported by factor analyses (Van Orden et al. 2012). The ITS posits that TB and PB are distinct, yet interrelated, constructs, supported by a moderately strong correlation between them (e.g., *r* = 0.57; Chu et al. 2017).

Despite initial psychometric support for the INQ‐15, prior findings have demonstrated inconsistencies in the degree to which TB and PB are related to SI; therefore, further psychometric evaluation of the INQ‐15 is needed to identify the source of these discrepancies as theoretical in nature or psychometric. Prior analyses using the INQ‐15 have shown that both TB and PB independently predict SI; however, PB has a stronger association with SI than TB (Chu et al. 2017). For example, among individuals who are psychiatrically hospitalized, bivariate correlations indicate TB and PB are both significantly associated with SI; however, after adjusting for depressive symptoms, only PB remains a significant predictor of current SI (Cero et al. 2015). Similarly, PB was a stronger correlate of SI compared to TB, followed by depressive symptoms and TB, respectively, among Veterans receiving treatment within an inpatient psychiatric unit (Monteith et al. 2013). Further, among individuals diagnosed with a mood disorder who were psychiatrically hospitalized for SI or a suicide attempt, PB but not TB was significantly associated with SI after adjusting for depressive symptoms, hopelessness, and mood disorder diagnosis (Taylor et al. 2016). Some have interpreted this pattern as suggesting that PB is a better predictor of SI than TB, deemphasizing the role of TB in the development of SI and, therefore, calling into question the theoretical underpinnings of the ITS (Chu et al. 2017).

However, the ITS and the broader empirical evidence emphasize TB as a central predictor of SI (Van Orden et al. 2010). For example, studies have shown significance in TB's association with other risk factors for SI (e.g., anxious and avoidant attachment style [Dienst et al. 2023], desire for death [O'Connor et al. 2017]). Findings from Mitchell et al. (2017) offer an alternative explanation for the discrepancies: both TB and PB are important predictors of SI, but TB may function differently in the context of PB. Specifically, this study found that both TB and PB were associated with increased distress due to SI when examined separately, but TB was no longer a significant predictor of SI distress when TB and PB were examined simultaneously. These results suggest TB may operate differently in the presence of PB, highlighting the need to clarify whether discrepancies in findings reflect psychometric issues with the INQ‐15. Thus, we aim to examine possible psychometric reasons for discrepancies in findings using the INQ‐15. Overall, should psychometric factors fail to account for these discrepancies, the findings would suggest the need for further examination of the theoretical assumptions regarding the relative roles of TB and PB in SI.

The factor structure of the INQ‐15 has been called into question in recent years, suggesting that its structure may be contributing to the mixed findings regarding the function of TB and PB (Quan et al. 2021). In fact, prior research suggests context effects (e.g., impact of the content of the item and the surrounding items in the survey) and serial order effects (e.g., changes in the answers to a survey question as a function of the position of the item in the questionnaire; Tourangeau et al. 2003) impact an individual's responses to items, influencing observed data patterns drawn from self‐report questionnaires (Longwell and Truax 2005; Steinberg 1994; Tourangeau et al. 2003). Tourangeau et al. (2003) posit that serial order effects and context effects may occur due to the priming (e.g., a set of beliefs or feelings triggered by an earlier question in the questionnaire) of certain underlying constructs. For example, the respondent naturally has more context to provide an interpretation to the later items in a survey. This is particularly notable, given the structure of the INQ‐15 (i.e., all PB questions are presented before TB questions).

Knowles (1988) demonstrated this idea, finding that items that appeared later in a self‐report questionnaire on a unidimensional construct were more related to the total score than items appearing earlier in the questionnaire. It is posited that this phenomenon could result from contextual effects or attentional focus during questionnaire completion (Steinberg 1994). Furthermore, Knowles (1988) and Hamilton and Shuminsky (1990) investigated the impact of serial order effects by creating alternative questionnaires that presented items in a rearranged order (i.e., each item was assessed in every possible position in the questionnaire). Their results indicated that the item and total score correlation increased as a function of serial position, and these effects were stronger when participants were in a state of decreased focus (Hamilton and Shuminsky 1990). Context and serial order effects can affect the interpretation of a single item or an entire construct, thereby influencing one's total score on a questionnaire.

Interestingly, the implications of serial order effects for the INQ‐15 are not straightforward. In the context of the INQ‐15, the first six items are loaded on the PB subscale (e.g., “These days, the people in my life would be better off if I were gone”), while the latter nine load onto the TB subscale (e.g., “These days, I rarely interact with people who care about me”; Van Orden et al. 2012). Although prior research has often found that PB demonstrates stronger associations with SI than TB (Chu et al. 2017), the ordering of items within the INQ‐15 could influence how respondents interpret and respond to both subscales. Given that all PB items are presented before all TB items, responses to either subscale may be influenced by contextual, attentional, or priming effects arising from the INQ‐15 structure. More broadly, the INQ‐15 structure may influence observed associations among TB, PB, and SI. Thus, it remains unclear whether discrepancies in the relative strength of TB and PB associations with SI reflect substantive differences between the constructs, measurement artifacts related to item sequencing, or some combination of both.

Overall, there appear to be two broad explanations for the discrepancies observed in the INQ‐15 literature: a true theoretical difference in how TB and PB relate to SI, or an artifact of psychometric and measurement factors (e.g., questionnaire structure, item sequencing, or factor structure). Thus, we aimed to investigate the role of various item sequences in the INQ‐15 factor structure, as well as the associations among TB, PB, and SI. We randomly assigned participants into three item sequence conditions: (1) PB‐TB (standard‐order: PB items, then TB items); (2) TB‐PB (reverse‐order: TB items, then PB items); and (3) random‐order of all items. Then, we explored how the psychometric properties of the INQ‐15 varied across conditions.

## 2 Method

### 2.1 Participants and Procedures

Participants (*N =* 600; 50.5% women; 66.8% White; M_age_ = 38.8 years) were recruited via Amazon Mechanical Turk (MTurk) and compensated for their participation in 2020. All participants were 18 years or older, English‐speaking, and lived within the United States. Past 6‐month SI was reported by 13.8% (*n* = 83), lifetime SI was reported by 34.2% (*n* = 205), and 11.2% (*n* = 67) reported a lifetime suicide attempt. See Table 1 for participant demographics.

**TABLE 1:** Demographic data and frequencies.

Demographic variables	Statistic
*N*	600
Age, M (SD)	38.75 (13.95)
Sex	
Male, *n* (%)	202 (33.7)
Female, *n* (%)	303 (50.5)
Race, *n* (%)	
White, *n* (%)	401 (66.8)
Hispanic, *n* (%)	65 (10.8)
Black/African American, *n* (%)	48 (8.0)
Asian/Asian American, *n* (%)	38 (6.3)
Native American, *n* (%)	8 (1.3)
Native Hawaiian/Pacific Islander, *n* (%)	2 (0.3)
Other, *n* (%)	13 (2.2)
Years of education, M (SD)	15.29 (2.28)
Housing status, *n* (%)	
Alone, *n* (%)	103 (17.2)
With family, *n* (%)	328 (54.7)
With friends, *n* (%)	43 (7.2)
With roommates, *n* (%)	7 (1.2)
No permanent living arrangements, *n* (%)	6 (1.0)
Other, *n* (%)	24 (4.0)
Partner status, *n* (%)	
Single, *n* (%)	137 (22.8)
Partnered, *n* (%)	99 (16.5)
Divorced, *n* (%)	34 (5.7)
Separated, *n* (%)	5 (0.8)
Married, *n* (%)	222 (37.0)
Widowed, *n* (%)	14 (2.3)
Employment status, *n* (%)	
Employed, *n* (%)	412 (68.7)
Unemployed, *n* (%)	99 (16.5)
Past 6‐month suicide ideation history, *n* (%)	83 (13.8)
Lifetime suicide ideation history, *n* (%)	205 (34.2)
Suicide attempt history, *n* (%)	67 (11.2)

Participants were provided with an information sheet and completed the survey measures anonymously. After consenting, participants were first randomly assigned to one of three conditions with different INQ‐15 item sequences and completed the INQ‐15 (standard‐order [PB‐TB], *n* = 192; reverse‐order [TB‐PB], *n* = 210; random‐order, *n* = 198). Subsequently, the other surveys were presented in a random sequence, with the demographic questionnaire always presented last. All participants were presented with national/international mental health and crisis resources at the end of the survey.

Given the potential for poor‐quality response data collected via online sampling, extensive data checks were conducted, including the removal of participants with nonsensical qualitative responses on the demographic questionnaire (e.g., providing unrelated, repeated answers for multiple questions) or missing the single INQ‐15 attention check item (i.e., *Please mark this response as “Yes”*) given the importance of the INQ‐15 to this study. Survey response times were also inspected, and participants were removed if their total response time appeared unreasonably short (e.g., completed 100% of the survey in less than the calculated minimum time, or completed a portion of the full survey in less than the calculated minimum time prorated by the percentage of the survey completed). In total, from 827 individuals who consented to the study, 227 participants were excluded (i.e., standard‐order, *n* = 80; reversed‐order, *n* = 66; random‐order, *n* = 81), and the remaining 600 participants were included in the analyses.

### 2.2 Measures

#### 2.2.1 Demographic Questionnaire

Participants completed a demographic questionnaire that assessed their background information (e.g., age, sex, race). This also included yes/no items about SI in the preceding 6 months (i.e., “Have you thought about or considered suicide (ending your own life) in the past six months?”), lifetime SI (i.e., “Have you ever thought about or considered suicide (ending your own life?)”), and lifetime suicide attempt (i.e., “Have you ever attempted suicide (you harmed yourself on purpose with at least some intention of ending your life)?”).

#### 2.2.2 Interpersonal Needs Questionnaire (INQ‐15)

The INQ‐15 (Van Orden et al. 2012) is a 15‐item self‐report assessment of recent feelings of PB and TB and has previously shown acceptable concurrent and predictive validity with SI (Van Orden et al. 2012). Items are rated on a 7‐point response scale ranging from 1 (*not at all true for me*) to 7 (*very true for me*). Items 7, 8, 10, 13, 14, and 15 (i.e., all items are on the TB subscale) are reverse‐scored. Scores are summed, and higher scores indicate greater PB (i.e., 6 items; PB score range: 6–42) and TB (i.e., 9 items; TB score range: 9–63). See the Supporting Information File (Tables S1–S6) for the INQ‐15 inter‐item correlations and item response frequencies. The INQ‐15 internal consistency values are provided in the results section.

#### 2.2.3 Positive and Negative Suicide Ideation Inventory (PANSI)

The PANSI (Osman et al. 1998) is a 14‐item self‐report inventory with two subscales: the PANSI‐Negative SI (PANSI‐NSI) and the PANSI‐Positive Ideation (PANSI‐PI); only the PANSI‐NSI was used in this study, as the PANSI‐PI captures items not explicitly tied to SI (e.g., hope, optimism). Items are rated on a 5‐point response scale ranging from 1 (*none of the time*) to 5 (*most of the time*), where higher scores on the PANSI‐NSI (i.e., 8 items, score range from 8 to 40) indicate greater negative thoughts related to SI. PANSI‐NSI has shown acceptable concurrent and predictive validity with SI (Muehlenkamp et al. 2005). For all conditions, the PANSI‐NSI subscale showed strong internal consistency (*α* = 0.95–0.98; *ω = 0*.95–0.98).

#### 2.2.4 Future Disposition Inventory‐24 (FDI‐24)

The FDI‐24 (Osman et al. 2010) is a 24‐item self‐report measure of hopelessness related to future‐related thoughts and feelings with strong psychometric properties (Bryan et al. 2013). The FDI‐24 consists of three 8‐item subscales (i.e., Positive Focus, Negative Focus, and Suicide Orientation) rated on a 5‐point Likert scale ranging from 1 (*not at all true of me*) to 5 (*extremely true of me*); in the current study, we only used the Suicide Orientation subscale. For all conditions, the FDI Suicide Orientation (FDI‐SO) subscale showed strong internal consistency (*α* = 0.96; *ω* = 0.97).

### 2.3 Data Analysis

We conducted confirmatory factor analyses (CFAs) using Mplus version 8.4 (Muthén and Muthén 1998–2026). We tested *Model 1* (i.e., the original, 2‐factor model) as our primary model. In addition, we tested three post hoc models aimed at addressing fit issues, including *Model 2* (i.e., 2‐factor model with correlated residuals for TB items 7, 8, 10, 13, 14, 15), *Model 3* (i.e., 2‐factor model with TB items 9, 11, and 12 removed), and *Model 4* (i.e., 2‐factor model with TB items 7, 8, 10, 13, 14, 15 removed). Given item response options were ordinal (i.e., responses ranged from 1 “not at all true for me” to 7 “very true for me”), INQ‐15 responses were specified as categorical, and the weighted least squares means and variances (WLSMV) estimator was used for all analyses. Additionally, WLSMV is resistant to normality violations (Muthén 1993), is preferred for certain types of missing data (Wang and Wang 2020), and allows fit indicators to be calculated for categorical indicators. No participants were missing all INQ‐15 data points; thus, we did not exclude any participants from analyses. There were also no individual INQ‐15 items that participants declined to answer; therefore, we did not code any data as missing.

CFAs were conducted to confirm the original INQ‐15 two‐factor structure (i.e., PB and TB; Van Orden et al. 2012; Model 1) for the standard‐order, the reverse‐order, and random‐order conditions. The variance of each latent variable (i.e., TB and PB) was constrained to one in all models, with all factor loadings freely estimated for each latent variable. The goodness of model fit for the CFA models was assessed using the Comparative Fit Index (CFI; Bentler 1990) and the Tucker–Lewis Index (TLI; Tucker and Lewis 1973). CFI and TLI values greater than 0.90 were considered to reflect an acceptable fit (Hu and Bentler 1999). In addition, the root mean square error of approximation (RMSEA) was reported with values less than 0.05, indicating excellent fit, and 0.05 to 0.08, indicating acceptable fit (Browne et al. 1993; Hu and Bentler 1999). Standardized root mean square residual (SRMR) is also a recommended model‐fit indicator for categorical CFA, with values less than 0.08 indicating good model fit (Hu and Bentler 1999).

## 3 Results

There were no significant demographic differences between the three conditions. Means and ranges for TB and PB were similar across conditions. Participants in the standard‐order condition had a PB mean of 13.69 (SD = 10.04; Md = 8), ranging from 6 to 42, and a TB mean of 28.13 (SD = 12.96; Md = 28), ranging from 9 to 63. Participants in the reverse‐order condition had a PB mean score of 13.49 (SD = 9.61; Md = 8), ranging from 6 to 42, and a TB mean score of 28.31 (SD = 11.94; Md = 28), ranging from 9 to 60. Participants in the random‐order condition had a PB mean score of 14.41 (SD = 9.95; Md = 9), ranging from 6 to 42, and a TB mean of 28.05 (SD = 12.41; Md = 28.5), ranging from 9 to 63. See the Supporting Information File (Tables S4–S6) for the INQ‐15 item‐level frequency data.

### 3.1 Construct Validity

Refer to Table 2 for the global fit indices of all models. The original two‐factor model (i.e., Model 1) in the standard‐order condition showed good model fit according to CFI (0.96) but poor model fit according to RMSEA (0.19) and SRMR (0.10). For Model 1 in the reverse‐order condition, CFI (0.96) and SRMR (0.07) indicated good model fit, whereas RMSEA (0.16) indicated poor model fit. Finally, Model 1 in the random‐order condition showed adequate model fit according to CFI (0.93) and poor model fit according to RMSEA (0.19) and SRMR (0.09). For all conditions, both PB and TB subscales showed good internal consistency (Cronbach's *α*: PB = 0.95–0.98; TB = 0.91–0.92; McDonald's *ω*: PB = 0.95–0.98; TB = 0.91–0.92; see Table 3).

**TABLE 2:** Model fit indices per condition.

	*χ* ^2^, df, *p*	RMSEA [90% CI], *p*	CFI	TLI	SRMR
Standard‐order condition					
Model 1	729.98, 89, < 0.001	0.19 [0.18, 0.21], < 0.001	0.96	0.95	0.10
Model 2	220.16, 74, < 0.001	0.10 [0.09, 0.12], < 0.001	0.99	0.99	0.03
Model 3	160.51, 53, < 0.001	0.10 [0.09, 0.12], < 0.001	0.99	0.99	0.03
Model 4	156.22, 26, < 0.001	0.16 [0.14, 0.19], < 0.001	0.99	0.99	0.03
Reverse‐order condition					
Model 1	556.22, 89, < 0.001	0.16 [0.15, 0.17], < 0.001	0.96	0.96	0.07
Model 2	205.82, 74, < 0.001	0.09 [0.08, 0.11], < 0.001	0.99	0.99	0.03
Model 3	141.94, 53, < 0.001	0.09 [0.07, 0.11], < 0.001	0.99	0.99	0.03
Model 4	91.92, 26, < 0.001	0.11 [0.09, 0.14], < 0.001	0.99	0.99	0.02
Random‐order condition					
Model 1	735.95, 89, < 0.001	0.19 [0.18, 0.21], < 0.001	0.93	0.92	0.09
Model 2	174.80, 74, < 0.001	0.08 [0.07, 0.10], < 0.001	0.99	0.99	0.03
Model 3	127.57, 53, < 0.001	0.08 [0.07, 0.10], 0.002	0.99	0.99	0.03
Model 4	62.64, 26, < 0.001	0.08 [0.06, 0.11], 0.02	0.99	0.99	0.02

*Note:* Model 1 = original, 2‐factor model; Model 2 = 2‐factor, correlated residuals for TB items 7, 8, 10, 13, 14, 15; Model 3 = 2‐factor, items 9, 11, and 12 removed; Model 4 = 2‐factor model TB items 7, 8, 10, 13, 14, 15 removed.

Abbreviations: CFI, Comparative Fit Index; CI, confidence interval; RMSEA, root mean square error approximation; SRMR, standardized root mean squared residual; TLI, Tucker–Lewis index.

**TABLE 3:** Descriptive statistics and bivariate correlations of study variables across conditions for models 1 and 3.

Standard‐order model 1	1	2	3	4
1. INQ PB	—	—	—	—
2. INQ TB	0.58*	—	—	—
3. FDI‐SO	0.80*	0.63*	—	—
4. PANSI‐NSI	0.77*	0.47*	0.86*	—
M	13.69	28.13	14.8	13.2
SD	10.04	12.96	8.1	8.16
Observed range	36	54	32	30
*α*	0.98	0.91	0.94	0.97
*ω*	0.98	0.91	0.94	0.97

Standard‐order model 3	5	6	7	8
5. INQ PB	—	—	—	—
6. INQ TB items removed	0.38*	—	—	—
7. FDI‐SO	0.80*	0.48*	—	—
8. PANSI‐NSI	0.77*	0.30*	0.86*	—
M	13.69	18.08	14.8	13.2
SD	10.04	9.08	8.1	8.16
Observed range	36	36	32	30
*α*	0.98	0.92	0.94	0.97
*ω*	0.98	0.92	0.94	0.97

Reverse‐order model 1	9	10	11	12
9. INQ PB	—	—	—	—
10. INQ TB	0.55*	—	—	—
11. FDI‐SO	0.80*	0.53*	—	—
12. PANSI‐NSI	0.72*	0.41*	0.84*	—
M	13.49	28.31	14.02	11.9
SD	9.61	11.94	8.02	6.99
Observed range	36	51	32	32
*α*	0.96	0.92	0.95	0.97
*ω*	0.96	0.92	0.95	0.97

Reverse‐order model 3	13	14	15	16
13. INQ PB	—	—	—	—
14. INQ TB items removed	0.41*	—	—	—
15. FDI‐SO	0.80*	0.41*	—	—
16. PANSI‐NSI	0.72*	0.31*	0.84*	—
M	13.49	17.99	14.02	11.9
SD	9.61	8.25	8.02	6.99
Observed range	36	33	32	32
*α*	0.96	0.93	0.95	0.97
*ω*	0.96	0.93	0.95	0.97

Random‐order model 1	17	18	19	20
17. INQ PB	—	—	—	—
18. INQ TB	0.60*	—	—	—
19. FDI‐SO	0.83*	0.57*	—	—
20. PANSI‐NSI	0.77*	0.43*	0.86*	—
M	14.41	28.05	14.89	13.24
SD	9.95	12.41	8.51	8.24
Observed range	36	54	29	31
*α*	0.95	0.91	0.95	0.97
*ω*	0.95	0.91	0.95	0.97

Random‐order model 3	21	22	23	24
21. INQ PB	—	—	—	—
22. INQ TB items removed	0.42*	—	—	—
23. FDI‐SO	0.83*	0.40*	—	—
24. PANSI‐NSI	0.77*	0.25*	0.86*	—
M	14.41	18.34	14.89	13.24
SD	9.95	8.56	8.51	8.24
Observed range	36	36	29	31
*α*	0.95	0.91	0.95	0.97
*ω*	0.95	0.91	0.95	0.97

*Note:* Model 1 = original, 2‐factor model; Model 3 = Interpersonal Needs Questionnaire (INQ), Thwarted Belonging, items 9, 11, and 12 removed.

Abbreviations: FDI‐SO, UTSA Future Dispositions Inventory Suicide Orientation subscale; INQ PB, Interpersonal Needs Questionnaire, Perceived Burden; INQ TB, Interpersonal Needs Questionnaire, Thwarted Belonging; PANSI‐NSI, Positive and Negative Suicide Ideation Inventory Negative Suicide Ideation subscale.

* *p* < 0.01.

Across all conditions, TB items 9, 11, and 12 were consistently problematic and raised model‐fit concerns based on modification indices, consistent with prior literature (Hill et al. 2015; Van Orden et al. 2012). Given that Model 1 across all three conditions had a poor to adequate fit, we conducted three post hoc analyses to improve model estimates. To account for method variance in Model 2, we correlated residuals for reverse‐coded items (i.e., TB items 7, 8, 10, 13, 14, 15), consistent with previous approaches (Asparouhov et al. 2015), improving model fit across all conditions. Model 2 in the standard‐order condition indicated good model fit according to CFI (0.99) and SRMR (0.03), although RMSEA (0.10) indicated poor fit. Model 2 in the reverse‐order condition indicated good model fit according to CFI (0.99) and SRMR (0.03), although RMSEA (0.09) was above the threshold for acceptable fit. Finally, Model 2 in the random‐order condition two‐factor model exhibited good model fit according to CFI (0.99), SRMR (0.03), and RMSEA (0.08).

For Model 3, we removed the 3 problematic items (i.e., TB items 9, 11, and 12), which similarly improved model fit. Model 3 for the standard‐order condition indicated good model fit according to CFI (0.99), SRMR (0.03), and RMSEA (0.10). Model 3 for the reverse‐order condition indicated good model fit according to CFI (0.99) and SRMR (0.03), although RMSEA (0.09) was above the threshold for acceptable fit. Finally, Model 3 for the random order two‐factor model exhibited good model fit according to CFI (0.99), SRMR (0.03), and RMSEA (0.08). For Model 3 where items 9, 11, and 12 were removed, Cronbach's *α* and McDonald's *ω* for PB (*α* = 0.95–0.98; *ω* = 0.95–0.98) and TB (*α* = 0.91–0.93; *ω* = 0.91–0.93; see Table 3) remained consistent across all conditions.

Finally, for Model 4, we removed all reverse‐coded TB items (i.e., items 7, 8, 10, 13, 14, 15). However, the model fit either remained consistent with or worsened relative to Models 2 and 3. Given that Models 2 and 3 retain most or all of the TB subscale items with similar model fit, we disregarded Model 4.

In summary, Model 3 is our preferred model, as it was more parsimonious, did not require correlated residual variances to improve model fit, and provided the best theoretical fit (i.e., weaker correlations between the TB and PB factors after problematic item removal; standard‐order, *r* = 0.33, *p* < 0.001; reverse‐order, *r* = 0.36, *p* < 0.001; random‐order, *r* = 0.36, *p* < 0.001). All factor loadings for Model 3 were strong (ranging from 0.71 to 0.94) and statistically significant at *p* < 0.05.

### 3.2 Concurrent Validity

In each of the three conditions and across models, positive correlations between the PANSI‐NSI (negative thoughts related to SI), the FDI‐SO (suicide orientation), PB, and TB subscales were significant (i.e., *p* < 0.001). However, across conditions, the associations between TB and PB, as well as between the INQ‐15 variables and SI‐related variables, did not differ notably across conditions. See Table 3 and the Supporting Information File (Table S7) for correlations and comparisions across all models.

## 4 Discussion

Given inconsistencies in the associations between TB, PB, and SI in the suicide literature (e.g., O'Connor et al. 2017; Taylor et al. 2016), we randomized participants (U.S. adult MTurk workers) into three INQ‐15 sequence conditions (i.e., standard‐order, reverse‐order, random‐order) to determine the impact of item sequencing on the associations. Overall, our findings support an absence of serial order effects (i.e., changes in the answers to a survey question as a function of the position of the item in the questionnaire) or context effects (i.e., impact of the content of the item and the surrounding items in the survey) within the INQ‐15. This suggests that the differential associations of TB and PB with SI are not attributable to item order within the INQ‐15. Thus, such patterns likely reflect true differences in associations rather than methodological artifacts.

However, across all conditions, TB items 9, 11, and 12 were consistently problematic and raised concerns about model fit, as indicated by the modification indices. Model fit issues related to items 9, 11, and 12 have also been identified in prior research (e.g., Hill et al. 2015; Upegui‐Arango et al. 2020; Van Orden et al. 2012). Potential contributors to the model fit concerns associated with TB items 9, 11, and 12 include differing item valences within the TB subscale and statistical artifacts associated with reverse coding. Specifically, the INQ‐15 PB subscale includes six negatively valenced items. Conversely, the TB subscale includes six positively valenced items (i.e., items 7, 8, 10, 13, 14, and 15) and three negatively valenced items (i.e., items 9, 11, and 12). Although items 9, 11, and 12 differ from the majority of the other INQ‐15 TB items, which are positively worded and non‐reverse‐coded, they are the only negatively worded, non‐reverse‐coded items within the TB subscale. Thus, model fit issues associated with these items may reflect a methodological artifact related to item valence and scoring differences (reverse scoring versus non‐reverse scoring) rather than substantive problems with the items themselves. Nevertheless, the content of these items also warrants consideration. Relative to the retained TB items, which primarily assess perceptions of being cared for, belonging, supportive relationships, and interpersonal closeness, items 9, 11, and 12 emphasize infrequent contact with caring others, social disconnection, and feeling like an outsider. Notably, these themes are highly consistent with the conceptualization of TB within the ITS, which emphasizes loneliness and unmet needs for social connection. As such, the findings raise the possibility that these items may capture a distinct aspect of TB not fully represented by the retained items. Future research should examine whether alternative factor structures better capture these distinctions and whether additional item development is needed to more comprehensively assess potential subdomains of TB. At the same time, future studies should evaluate whether the observed findings are better explained by item valence and wording effects by testing revised versions of the INQ in which all items within each subscale are consistently valenced.

Furthermore, correlating the residuals for items 7, 8, 10, 13, 14, 15 (i.e., to account for residual variance due to measurement error and variance uniquely associated with the reverse‐coded, positively valenced items in the TB subscale) improved model fit (Model 2); however, removing these items (i.e., Model 4) did not improve the model fit for any conditions. Removing only the problematic items (9, 11, and 12) from the TB scale (Model 3) similarly improved model fit across conditions, yielding model fit comparable to that of Model 2. Of note, for Model 3, the correlations between the TB and PB factors were weaker than those reported in the literature (e.g., Van Orden et al. 2008). Thus, the factors resulting from Model 3 may be more distinct and could help address multicollinearity that affects the testing of the ITS, thereby potentially resolving the discrepant findings regarding the roles of TB and PB as predictors of SI (Mitchell et al. 2020). This possibility should be tested in future research.

If items 9, 11, and 12 reflect a distinct aspect of TB related to social isolation and alienation, the improved model fit observed following their removal may not necessarily indicate that these experiences are unimportant. Rather, the findings may raise the possibility that the current operationalization of TB within the INQ‐15 combines related, but potentially distinguishable, interpersonal experiences into a single factor. Future research should examine whether alternative factor structures, including models that separate perceived interpersonal connectedness from social isolation and alienation, provide a better representation of the construct. Such work may also inform future item development efforts aimed at more comprehensively assessing the potentially multidimensional nature of TB.

### 4.1 Implications

The results of this study have several implications for research and clinical practice. In research contexts, results suggest that various item sequences are acceptable with the INQ‐15, allowing for flexibility in administration. Results also highlight the need to further examine the psychometric properties of the INQ‐15 across samples, with particular attention to items 9, 11, and 12 on the TB subscale. Further research may support revising the INQ‐15 to enhance its psychometric properties (e.g., by modifying, replacing, or removing items that compromise model fit across conditions). Researchers should consider examining the factor structure of the INQ‐15 in their data before proceeding with other analyses. They could also consider comparing results between the INQ‐15 with all 15 items included and the INQ‐15 after removing items 9, 11, and 12. Future research may also examine a revised version of the INQ‐15 in which all items within each subscale are consistently valenced (e.g., negatively or positively). This may help address item‐level inconsistencies identified in the current study. Regarding clinical implications, these results suggest that clinicians should be aware of the potential limitations of items 9, 11, and 12 on the INQ‐15, given consistent fit problems with those items within these data. Clinicians should interpret responses to items 9, 11, and 12 with caution, given their consistent contribution to model fit concerns in the current and prior studies. Additional research is needed before definitive recommendations regarding their removal can be made.

### 4.2 Limitations

Our results should be interpreted in the context of several limitations. First, participants were limited to U.S.‐residing Amazon MTurk workers, and only one‐third of participants reported lifetime SI. Results may vary in other populations, such as clinical populations, participants residing in different countries, or those recruited through alternative avenues. Second, some response categories had sparse (< 5%) endorsement. We chose not to collapse sparse response categories due to inconsistencies in which categories were sparse across the three item‐sequence conditions. However, collapsing sparse responses into fewer response categories may be advantageous in some circumstances (DiStefano et al. 2021). Third, SI‐related measures (i.e., PANSI‐NSI, FDI‐SO) used to examine concurrent validity assess a range of experiences related to SI, including active SI, passive SI, and specific reasons for SI; more specific relations between TB, PB, and facets of SI were not examined. Fourth, while post hoc efforts were made to ensure data quality for the current study (i.e., removing participants with nonsensical qualitative responses, participants who missed an attention‐check item, and participants with implausibly fast completion times), respondents may have been prone to careless responding, and the methods available to the authors to identify careless responding were limited (Ward and Meade 2023). Future research should replicate our analyses in other samples.

## 5 Conclusions

Our results add to a growing body of literature suggesting greater attention is needed to the factor structure of the INQ‐15. Importantly, the factor structure of the INQ‐15 did not differ across three item‐sequencing conditions (standard‐order, reversed‐order, or random‐order), indicating the absence of serial‐order or context effects within the INQ‐15. This suggests that subscale ordering within the INQ‐15 does not impact participant responding. This supports the possibility of differential associations between TB and PB, with SI reflecting actual differences in their relations with SI rather than item‐sequence methodological artifacts. However, items 9, 11, and 12 on the TB subscale consistently contributed to poor model fit, consistent with prior studies. Several alternative models improved model fit, suggesting that the factor structure of the INQ‐15 be revisited and further tested, and that a shortened INQ, after removing the problematic items, may have better psychometric properties. Given the current findings, researchers may wish to examine the psychometric consequences of removing items 9, 11, and 12 from the INQ‐15 and compare results across alternative scoring approaches. However, because these items may capture potentially meaningful aspects of TB related to social isolation and alienation, additional research is needed before definitive recommendations regarding their removal can be made.

## Author Contributions

**Julianne E. Cary:** writing – original draft, writing – review and editing, formal analysis. **Sean M. Mitchell:** conceptualization, investigation, funding acquisition, writing – review and editing, methodology, data curation, supervision, resources, writing – original draft. **Kirsten Christensen:** writing – original draft, writing – review and editing, formal analysis. **Sarah Sparks:** writing – review and editing, writing – original draft, formal analysis, conceptualization, investigation. **Sarah E. Victor:** writing – review and editing. **Sarah L. Brown:** conceptualization, investigation, writing – review and editing, methodology, funding acquisition.

## Funding

Time for this research was supported, in part, by funding from the National Institute of Mental Health (L30 MH120575; F32 MH126527; F31 MH136767).

## Disclosure

The content is solely the responsibility of the authors and does not necessarily represent the official views of the funding agencies.

## Ethics Statement

The Texas Tech University Institutional Review Board approved all procedures.

## Consent

All participants provided informed consent to participate in this research.

## Conflicts of Interest

The authors declare no conflicts of interest.

## Supporting information


**Table S1:** Standard order Interpersonal Needs Questionnaire (INQ) inter‐item correlations (*N* = 192).
**Table S2:** Reverse order Interpersonal Needs Questionnaire (INQ) inter‐item correlations (*N* = 210).
**Table S3:** Random order Interpersonal Needs Questionnaire inter‐item correlations (*N* = 198).
**Table S4:** Standard order original structure model (*N* = 192) responses to each item of the Interpersonal Needs Questionnaire that were used in confirmatory factor analyses.
**Table S5:** Reverse order original structure model (*N* = 210) responses to each item of the Interpersonal Needs Questionnaire that were used in confirmatory factor analyses.
**Table S6:** Random order original structure model (*N* = 198) responses to each item of the Interpersonal Needs Questionnaire that were used in confirmatory factor analyses.
**Table S7:** Correlations between PB, TB, and suicide ideation‐related variables for standard‐order, reverse‐order, and random‐order model 3 conditions.

## Data Availability

This study was not pre‐registered. The data and code are not publicly available but are available upon request by emailing the corresponding author.
