# Supplementary material for: Examining the Role of Item Sequencing of the Interpersonal Needs Questionnaire‐15
Source: Suicide Life Threat Behav. 2026 Aug 16;56(4):e70133. doi: 10.1111/sltb.70133 (PMC13478541; doi:10.1111/sltb.70133)
Supplement: Supplementary file 1 [file SLTB-56-0-s001.docx]

**Examining the Role of Item Sequencing of the Interpersonal Needs Questionnaire-15**

***Supporting Information File***

**Table S1**

*Standard Order Interpersonal Needs Questionnaire (INQ) Inter-Item Correlations (N = 192)*

| **INQ Item Number** | **1** | **2** | **3** | **4** | **5** | **6** | **7** | **8** | **9** | **10** | **11** | **12** | **13** | **14** |
| --- | --- | --- | --- | --- | --- | --- | --- | --- | --- | --- | --- | --- | --- | --- |
| 1. Item 1 | - | - | - | - | - | - | - | - | - | - | - | - | - | - |
| 2. Item 2 | .88 | - | - | - | - | - | - | - | - | - | - | - | - | - |
| 3. Item 3 | .84 | .86 | - | - | - | - | - | - | - | - | - | - | - | - |
| 4. Item 4 | .89 | .88 | .84 | - | - | - | - | - | - | - | - | - | - | - |
| 5. Item 5 | .86 | .89 | .87 | .90 | - | - | - | - | - | - | - | - | - | - |
| 6. Item 6 | .88 | .86 | .88 | .86 | .84 | - | - | - | - | - | - | - | - | - |
| 7. Item 7 | .38 | .35 | .35 | .38 | .34 | .39 | - | - | - | - | - | - | - | - |
| 8. Item 8 | .32 | .31 | .38 | .29 | .28 | .40 | .71 | - | - | - | - | - | - | - |
| 9. Item 9 | .69 | .73 | .68 | .66 | .71 | .70 | .43 | .36 | - | - | - | - | - | - |
| 10. Item 10 | .25 | .23 | .29 | .27 | .27 | .33 | .67 | .75 | .31 | - | - | - | - | - |
| 11. Item 11 | .60 | .57 | .64 | .58 | .56 | .69 | .44 | .54 | .61 | .53 | - | - | - | - |
| 12. Item 12 | .56 | .57 | .65 | .55 | .54 | .66 | .40 | .51 | .60 | .45 | .83 | - | - | - |
| 13. Item 13 | .26 | .32 | .32 | .32 | .30 | .32 | .56 | .59 | .33 | .64 | .42 | .36 | - | - |
| 14. Item 14 | .23 | .25 | .31 | .21 | .19 | .30 | .54 | .70 | .35 | .69 | .51 | .48 | .68 | - |
| 15. Item 15 | .31 | .30 | .30 | .28 | .26 | .31 | .63 | .66 | .35 | .64 | .46 | .41 | .69 | .73 |

*Note*. Perceived Burdensomeness subscale includes items 1 – 6; Thwarted Belongingness subscale includes items 7 – 15; All correlations were significant with *p* < .001.

**Table S2**

*Reverse Order Interpersonal Needs Questionnaire (INQ) Inter-Item Correlations (N = 210)*

| **INQ Item Number** | **1** | **2** | **3** | **4** | **5** | **6** | **7** | **8** | **9** | **10** | **11** | **12** | **13** | **14** |
| --- | --- | --- | --- | --- | --- | --- | --- | --- | --- | --- | --- | --- | --- | --- |
| 1. Item 1 | - | - | - | - | - | - | - | - | - | - | - | - | - | - |
| 2. Item 2 | .84 | - | - | - | - | - | - | - | - | - | - | - | - | - |
| 3. Item 3 | .73 | .80 | - | - | - | - | - | - | - | - | - | - | - | - |
| 4. Item 4 | .77 | .84 | .79 | - | - | - | - | - | - | - | - | - | - | - |
| 5. Item 5 | .80 | .84 | .77 | .83 | - | - | - | - | - | - | - | - | - | - |
| 6. Item 6 | .81 | .85 | .86 | .81 | .86 | - | - | - | - | - | - | - | - | - |
| 7. Item 7 | .31 | .32 | .44 | .30 | .29 | .34 | - | - | - | - | - | - | - | - |
| 8. Item 8 | .28 | .36 | .44 | .25 | .31 | .39 | .70 | - | - | - | - | - | - | - |
| 9. Item 9 | .50 | .48 | .47 | .40 | .41 | .43 | .34 | .29 | - | - | - | - | - | - |
| 10. Item 10 | .20 | .25 | .33 | .19 | .25 | .29 | .68 | .64 | .28 | - | - | - | - | - |
| 11. Item 11 | .54 | .60 | .64 | .49 | .54 | .59 | .54 | .65 | .55 | .58 | - | - | - | - |
| 12. Item 12 | .55 | .56 | .58 | .46 | .46 | .54 | .43 | .52 | .42 | .45 | .75 | - | - | - |
| 13. Item 13 | .35 | .38 | .48 | .29 | .28 | .40 | .73 | .64 | .34 | .71 | .59 | .46 | - | - |
| 14. Item 14 | .28 | .33 | .43 | .24 | .27 | .36 | .68 | .69 | .39 | .72 | .64 | .52 | .78 | - |
| 15. Item 15 | .32 | .37 | .49 | .25 | .30 | .38 | .65 | .67 | .31 | .63 | .63 | .53 | .71 | .75 |

*Note*. Perceived Burdensomeness subscale includes items 1 – 6; Thwarted Belongingness subscale includes items 7 – 15; All correlations were significant with *p* < .001.

**Table S3**

*Random Order Interpersonal Needs Questionnaire Inter-Item Correlations (N = 198)*

| **Item Number** | **1** | **2** | **3** | **4** | **5** | **6** | **7** | **8** | **9** | **10** | **11** | **12** | **13** | **14** |
| --- | --- | --- | --- | --- | --- | --- | --- | --- | --- | --- | --- | --- | --- | --- |
| 1. Item 1 | - | - | - | - | - | - | - | - | - | - | - | - | - | - |
| 2. Item 2 | .81** | - | - | - | - | - | - | - | - | - | - | - | - | - |
| 3. Item 3 | .79** | .69** | - | - | - | - | - | - | - | - | - | - | - | - |
| 4. Item 4 | .83** | .74** | .77** | - | - | - | - | - | - | - | - | - | - | - |
| 5. Item 5 | .80** | .75** | .77** | .75** | - | - | - | - | - | - | - | - | - | - |
| 6. Item 6 | .83** | .78** | .79** | .75** | .77** | - | - | - | - | - | - | - | - | - |
| 7. Item 7 | .46** | .41** | .46** | .35** | .45** | .48** | - | - | - | - | - | - | - | - |
| 8. Item 8 | .32** | .29** | .38** | .27** | .32** | .36** | .65** | - | - | - | - | - | - | - |
| 9. Item 9 | .67** | .60** | .61** | .64** | .66** | .63** | .44** | .39** | - | - | - | - | - | - |
| 10. Item 10 | .29** | .26** | .31** | .23** | .29** | .31** | .65** | .68** | .41** | - | - | - | - | - |
| 11. Item 11 | .68** | .60** | .62** | .56** | .60** | .65** | .48** | .54** | .71** | .53** | - | - | - | - |
| 12. Item 12 | .54** | .48** | .55** | .52** | .48** | .56** | .41** | .54** | .57** | .48** | .71** | - | - | - |
| 13. Item 13 | .28** | .27** | .33** | .21* | .30** | .33** | .68** | .71** | .40** | .74** | .49** | .39** | - | - |
| 14. Item 14 | .32** | .28** | .35** | .27** | .28** | .34** | .69** | .73** | .44** | .65** | .52** | .48** | .70** | - |
| 15. Item 15 | .23** | .18* | .30** | .24** | .29** | .26** | .49** | .55** | .42** | .54** | .38** | .40** | .53** | .56** |

*Note*. Perceived Burdensomeness subscale includes items 1 – 6; Thwarted Belongingness subscale includes items 7 – 15; ^**^*p* < .01, ^*^*p* < .05.

**Table S4**

*Standard Order Original Structure Model (N = 192) Responses to Each Item of the Interpersonal Needs Questionnaire that Were Used in Confirmatory Factor Analyses*

| **Item** | **Response Options** | | | | | | |
| --- | --- | --- | --- | --- | --- | --- | --- |
|  | **1**  **(Not at all**  **true for me)** | **2** | **3** | **4**  **(Somewhat**  **true for me)** | **5** | **6** | **7 (Very**  **true for me)** |
| Item 1: These days, the people in my life would be better off if I were gone | | | | | |  |  |
| *n* | 104 | 25 | 20 | 19 | 9 | 8 | 7 |
| % | 54.2 | 13.0 | 10.4 | 9.9 | 4.7 | 4.2 | 3.6 |
| Item 2: These days, the people in my life would be happier without me | | | | | |  |  |
| *n* | 102 | 26 | 22 | 16 | 12 | 8 | 6 |
| % | 53.1 | 13.5 | 11.5 | 8.3 | 6.3 | 4.2 | 3.1 |
| Item 3: These days, I think I am a burden on society | | | |  |  |  |  |
| *n* | 98 | 25 | 15 | 24 | 9 | 12 | 9 |
| % | 51.0 | 13.0 | 7.8 | 12.5 | 4.7 | 6.3 | 4.7 |
| Item 4: These days, I think my death would be a relief to the people in my life | | | | | | | |
| *n* | 123 | 16 | 12 | 17 | 10 | 7 | 7 |
| % | 64.1 | 8.3 | 6.3 | 8.9 | 5.2 | 3.6 | 3.6 |
| Item 5: These days, I think the people in my life wish they could be rid of me | | | | | |  |  |
| *n* | 109 | 26 | 16 | 14 | 10 | 9 | 8 |
| % | 56.8 | 13.5 | 8.3 | 7.3 | 5.2 | 4.7 | 4.2 |
| Item 6: These days, I think I make things worse for the people in my life | | | | | |  |  |
| *n* | 92 | 32 | 14 | 26 | 12 | 6 | 10 |
| % | 47.9 | 16.7 | 7.3 | 13.5 | 6.3 | 3.1 | 5.2 |
| Item 7: These days, other people care about me | | | | |  |  |  |
| *n* | 53 | 50 | 35 | 29 | 8 | 7 | 10 |
| % | 27.6 | 26.0 | 18.2 | 15.1 | 4.2 | 3.6 | 5.2 |
| Item 8: These days, I feel like I belong | | | |  |  |  |  |
| *n* | 38 | 43 | 39 | 32 | 12 | 12 | 16 |
| % | 19.8 | 22.4 | 20.3 | 16.7 | 6.3 | 6.3 | 8.3 |
| Item 9: These days, I rarely interact with people who care about me | | | | | |  |  |
| *n* | 69 | 33 | 21 | 28 | 14 | 11 | 16 |
| % | 35.9 | 17.2 | 10.9 | 14.6 | 7.3 | 5.7 | 8.3 |
| Item 10: These days, I am fortunate to have many caring and supportive friends | | | | | |  |  |
| *n* | 49 | 45 | 31 | 32 | 10 | 11 | 14 |
| % | 25.5 | 23.4 | 16.1 | 16.7 | 5.2 | 5.7 | 7.3 |
| Item 11: These days, I feel disconnected from other people | | | | | | | |
| *n* | 49 | 30 | 18 | 26 | 30 | 19 | 20 |
| % | 25.5 | 15.6 | 9.4 | 13.5 | 15.6 | 9.9 | 10.4 |
| Item 12: These days, I often feel like an outsider in social gatherings | | | | | | | |
| *n* | 45 | 23 | 26 | 32 | 20 | 19 | 27 |
| % | 23.4 | 12.0 | 13.5 | 16.7 | 10.4 | 9.9 | 14.1 |
| Item 13: These days, I feel that there are people I can turn to in times of need | | | | | | | |
| *n* | 47 | 46 | 39 | 26 | 9 | 12 | 13 |
| % | 24.5 | 24.0 | 20.3 | 13.5 | 4.7 | 6.3 | 6.8 |
| Item 14: These days, I am close to other people | | | | | | | |
| *n* | 42 | 39 | 38 | 29 | 15 | 14 | 15 |
| % | 21.9 | 20.3 | 19.8 | 15.1 | 7.8 | 7.3 | 7.8 |
| Item 15: These days, I have at least one satisfying interaction every day | | | | | | | |
| *n* | 48 | 40 | 35 | 36 | 12 | 8 | 13 |
| % | 25.0 | 20.8 | 18.2 | 18.8 | 6.3 | 4.2 | 6.8 |

**Table S5**

*Reverse Order Original Structure Model (N = 210) Responses to Each Item of the Interpersonal Needs Questionnaire that Were Used in Confirmatory Factor Analyses*

| **Response Options** | | | | | | | |
| --- | --- | --- | --- | --- | --- | --- | --- |
| **Item** | **1**  **(Not at all true for me)** | **2** | **3** | **4**  **(Somewhat true for me)** | **5** | **6** | **7**  **(Very true for me)** |
| Item 1: These days, the people in my life would be better off if I were gone | | | | | |  |  |
| *n* | 115 | 23 | 13 | 19 | 21 | 12 | 7 |
| % | 54.8 | 11.0 | 6.2 | 9.0 | 10.0 | 5.7 | 3.3 |
| Item 2: These days, the people in my life would be happier without me | | | | | |  |  |
| *n* | 114 | 25 | 20 | 20 | 15 | 11 | 5 |
| % | 54.3 | 11.9 | 9.5 | 9.5 | 7.1 | 5.2 | 2.4 |
| Item 3: These days, I think I am a burden on society | | | | | | | |
| *n* | 107 | 29 | 18 | 27 | 16 | 8 | 5 |
| % | 51.0 | 13.8 | 8.6 | 12.9 | 7.6 | 3.8 | 2.4 |
| Item 4: These days, I think my death would be a relief to the people in my life | | | | | |  |  |
| *n* | 130 | 26 | 12 | 19 | 8 | 11 | 4 |
| % | 61.9 | 12.4 | 5.7 | 9.0 | 3.8 | 5.2 | 1.9 |
| Item 5: These days, I think the people in my life wish they could be rid of me | | | | | |  |  |
| *n* | 126 | 26 | 12 | 22 | 8 | 12 | 4 |
| % | 60.0 | 12.4 | 5.7 | 10.5 | 3.8 | 5.7 | 1.9 |
| Item 6: These days, I think I make things worse for the people in my life | | | | | |  |  |
| *n* | 115 | 27 | 13 | 16 | 22 | 11 | 6 |
| % | 54.8 | 12.9 | 6.2 | 7.6 | 10.5 | 5.2 | 2.9 |
| Item 7: These days, other people care about me | | | | |  |  |  |
| *n* | 51 | 56 | 40 | 41 | 11 | 8 | 3 |
| % | 24.3 | 26.7 | 19.0 | 19.5 | 5.2 | 3.8 | 1.4 |
| Item 8: These days, I feel like I belong | | | |  |  |  |  |
| *n* | 34 | 48 | 45 | 43 | 14 | 18 | 8 |
| % | 16.2 | 22.9 | 21.4 | 20.5 | 6.7 | 8.6 | 3.8 |
| Item 9: These days, I rarely interact with people who care about me | | | | | |  |  |
| *n* | 46 | 41 | 40 | 28 | 11 | 28 | 16 |
| % | 21.9 | 19.5 | 19.0 | 13.3 | 5.2 | 13.3 | 7.6 |
| Item 10: These days, I am fortunate to have many caring and supportive friends | | | | | | | |
| *n* | 43 | 49 | 33 | 40 | 28 | 9 | 8 |
| % | 20.5 | 23.3 | 15.7 | 19.0 | 13.3 | 4.3 | 3.8 |
| Item 11: These days, I feel disconnected from other people | | | | | | | |
| *n* | 45 | 41 | 23 | 30 | 33 | 25 | 13 |
| % | 21.4 | 19.5 | 11.0 | 14.3 | 15.7 | 11.9 | 6.2 |
| Item 12: These days, I often feel like an outsider in social gatherings | | | | | | | |
| *n* | 32 | 43 | 27 | 38 | 35 | 20 | 15 |
| % | 15.2 | 20.5 | 12.9 | 18.1 | 16.7 | 9.5 | 7.1 |
| Item 13: These days, I feel that there are people I can turn to in times of need | | | | | | | |
| *n* | 52 | 56 | 40 | 29 | 22 | 8 | 3 |
| % | 24.8 | 26.7 | 19.0 | 13.8 | 10.5 | 3.8 | 1.4 |
| Item 14: These days, I am close to other people | | | | | | | |
| *n* | 35 | 41 | 55 | 33 | 25 | 15 | 6 |
| % | 16.7 | 19.5 | 26.2 | 15.7 | 11.9 | 7.1 | 2.9 |
| Item 15: These days, I have at least one satisfying interaction every day | | | | | | | |
| *n* | 46 | 51 | 33 | 35 | 27 | 12 | 6 |
| % | 21.9 | 24.3 | 15.7 | 16.7 | 12.9 | 5.7 | 2.9 |

**Table S6**

*Random Order Original Structure Model (N = 198) Responses to Each Item of the Interpersonal Needs Questionnaire that Were Used in Confirmatory Factor Analyses*

| **Response Options** | | | | | | | |
| --- | --- | --- | --- | --- | --- | --- | --- |
| **Item** | **1**  **(Not at all true for me)** | **2** | **3** | **4**  **(Somewhat true for me)** | **5** | **6** | **7**  **(Very true for me)** |
| Item 1: These days, the people in my life would be better off if I were gone | | | | | |  |  |
| *n* | 111 | 19 | 10 | 21 | 19 | 12 | 6 |
| % | 56.1 | 9.6 | 5.1 | 10.6 | 9.6 | 6.1 | 3.0 |
| Item 2: These days, the people in my life would be happier without me | | | | | |  |  |
| *n* | 103 | 25 | 20 | 19 | 16 | 9 | 6 |
| % | 52.0 | 12.6 | 10.1 | 9.6 | 8.1 | 4.5 | 3.0 |
| Item 3: These days, I think I am a burden on society | | | | | | | |
| *n* | 104 | 19 | 13 | 19 | 19 | 15 | 9 |
| % | 52.5 | 9.6 | 6.6 | 9.6 | 9.6 | 7.6 | 4.5 |
| Item 4: These days, I think my death would be a relief to the people in my life | | | | | |  |  |
| *n* | 120 | 11 | 15 | 19 | 17 | 12 | 4 |
| % | 60.6 | 5.6 | 7.6 | 9.6 | 8.6 | 6.1 | 2.0 |
| Item 5: These days, I think the people in my life wish they could be rid of me | | | | | |  |  |
| *n* | 110 | 22 | 11 | 25 | 12 | 12 | 6 |
| % | 55.6 | 11.1 | 5.6 | 12.6 | 6.1 | 6.1 | 3.0 |
| Item 6: These days, I think I make things worse for the people in my life | | | | | |  |  |
| *n* | 92 | 29 | 13 | 28 | 20 | 11 | 5 |
| % | 46.5 | 14.6 | 6.6 | 14.1 | 10.1 | 5.6 | 2.5 |
| Item 7: These days, other people care about me | | | | |  |  |  |
| *n* | 57 | 51 | 28 | 34 | 13 | 8 | 7 |
| % | 28.8 | 25.8 | 14.1 | 17.2 | 6.6 | 4.0 | 3.5 |
| Item 8: These days, I feel like I belong | | | |  |  |  |  |
| *n* | 32 | 41 | 39 | 35 | 25 | 10 | 16 |
| % | 16.2 | 20.7 | 19.7 | 17.7 | 12.6 | 5.1 | 8.1 |
| Item 9: These days, I rarely interact with people who care about me | | | | | |  |  |
| *n* | 71 | 33 | 24 | 32 | 16 | 12 | 10 |
| % | 35.9 | 16.7 | 12.1 | 16.2 | 8.1 | 6.1 | 5.1 |
| Item 10: These days, I am fortunate to have many caring and supportive friends | | | | | |  |  |
| *n* | 46 | 44 | 35 | 35 | 15 | 14 | 9 |
| % | 23.2 | 22.2 | 17.7 | 17.7 | 7.6 | 7.1 | 4.5 |
| Item 11: These days, I feel disconnected from other people | | | | | | | |
| *n* | 63 | 22 | 27 | 32 | 17 | 18 | 19 |
| % | 31.8 | 11.1 | 13.6 | 16.2 | 8.6 | 9.1 | 9.6 |
| Item 12: These days, I often feel like an outsider in social gatherings | | | | | | | |
| *n* | 41 | 22 | 28 | 40 | 27 | 23 | 17 |
| % | 20.7 | 11.1 | 14.1 | 20.2 | 13.6 | 11.6 | 8.6 |
| Item 13: These days, I feel that there are people I can turn to in times of need | | | | | | | |
| *n* | 54 | 49 | 38 | 27 | 13 | 12 | 5 |
| % | 27.3 | 24.7 | 19.2 | 13.6 | 6.6 | 6.1 | 2.5 |
| Item 14: These days, I am close to other people | | | | | | | |
| *n* | 38 | 39 | 31 | 43 | 21 | 17 | 9 |
| % | 19.2 | 19.7 | 15.7 | 21.7 | 10.6 | 8.6 | 4.5 |
| Item 15: These days, I have at least one satisfying interaction every day | | | | | | | |
| *n* | 36 | 50 | 36 | 36 | 13 | 17 | 10 |
| % | 18.2 | 25.3 | 18.2 | 18.2 | 6.6 | 8.6 | 5.1 |

**Table S7**

*Comparisons of Correlations Between PB, TB, and Suicide Ideation-Related Variables Across Standard-Order, Reverse-Order, and Random-Order Conditions for Model 3*

| **INQ PB/INQ TB** | Standard-Order | Reverse-Order |
| --- | --- | --- |
| Reverse-Order | .362 | - |
| Random-Order | .32 | .452 |
| **INQ PB/FDI-SO** | Standard-Order | Reverse-Order |
| Reverse-Order | .50 | - |
| Random-Order | .19 | .185 |
| **INQ TB/FDI-SO** | Standard-Order | Reverse-Order |
| Reverse-Order | .193 | - |
| Random-Order | .165 | .452 |
| **INQ PB/PANSI-NSI** | Standard-Order | Reverse-Order |
| Reverse-Order | .131 | - |
| Random-Order | .50 | .129 |
| **INQ TB/PANSI-NSI** | Standard-Order | Reverse-Order |
| Reverse-Order | .456 | - |
| Random-Order | .298 | .257 |
| **FDI-SO/PANSI-NSI** | Standard-Order | Reverse-Order |
| Reverse-Order | .237 | - |
| Random-Order | .50 | .235 |

*Note.* Model 3 = Interpersonal Needs Questionnaire, Thwarted Belonging, items 9, 11, and 12 removed; INQ PB = Interpersonal Needs Questionnaire, Perceived Burdensomeness; INQ TB = Interpersonal Needs Questionnaire, Thwarted Belonging; FDI-SO = UTSA Future Dispositions Inventory Suicide Orientation subscale; PANSI-NSI = Positive and Negative Suicide Ideation Inventory Negative Suicide Ideation subscale. Values presented in the table are p values for pairwise comparisons of correlations across conditions. All comparisons were nonsignificant, *p* > .05.
